# Synthesis, Docking and Biological Evaluation of a Novel Class of Imidazothiazoles as IDO1 Inhibitors

**DOI:** 10.3390/molecules24101874

**Published:** 2019-05-15

**Authors:** Marta Serafini, Enza Torre, Silvio Aprile, Alberto Massarotti, Silvia Fallarini, Tracey Pirali

**Affiliations:** Department of Pharmaceutical Sciences, Università del Piemonte Orientale, Largo Donegani 2, 28100 Novara, Italy; marta.serafini@uniupo.it (M.S.); enza.torre@uniupo.it (E.T.); silvio.aprile@uniupo.it (S.A.); alberto.massarotti@uniupo.it (A.M.); silvia.fallarini@uniupo.it (S.F.)

**Keywords:** indoleamine 2,3-dioxygenase 1, click chemistry, imidazothiazoles, docking

## Abstract

IDO1, a key dioxygenase in tryptophan-kynurenine metabolism, appeared in the last 10 years at the vanguard of druggable targets in cancer therapy due to its well-established role both in immune escape and inflammatory neovascularization. Among the pool of IDO1 inhibitors that have entered clinical trials, none have reached approval. The identification of novel inhibitors endowed with better clinical profile, together with the further comprehension of the interactions with residues in IDO1 active site, are still a need. In this context, we have synthesized a novel class of imidazothiazole derivatives as IDO1 inhibitors and identified three compounds with inhibitory potency in the low micromolar range. This report strengthens the role played by pocket C in the active site of IDO1, providing novel directions in the design of IDO1 inhibitors.

## 1. Introduction

The tryptophan to kynurenine catabolism and the dioxygenases that catalyse the first and rate-limiting step along the kynurenine pathway play a crucial role in the pathological immune escape [1,2,3]. Depletion of tryptophan and the consequent increase in kynurenines in the tumour microenvironment have been shown to enhance inflammation and lead to an immune-permissive milieu [4] as well as to promote neo-angiogenesis [5]. Among the three different isoforms of dioxygenases, IDO1, IDO2 and TDO, the former has appeared at centre-stage in the cancer field with hundreds of IDO1 inhibitors discovered in the past [6]. 

In the last decades checkpoint inhibitors (i.e., anti PD-1/PD-L1) have revolutionized the scenario of cancer immunotherapy [7]. Nevertheless, it is becoming apparent that molecules able to boost the effects of cancer immunotherapy can potentially provide superior response rates, considering that a significant proportion of patients do not respond to these drugs. Great hopes have been placed on IDO1 inhibitors with five different molecules (indoximod, navoximod, epacadostat, BMS-986205, PF-06840003) that have entered clinical trials. Currently, navoximod and PF-06840003 are in Phase I trials, while indoximod, BMS-986205 and epacadostat have reached Phase III trials for the treatment of different types of cancers (e.g., melanoma, solid tumors, malignant glioma) [6]. 

In 2018 the report at the ASCO Annual Meeting that epacadostat failed to show a clinical benefit in combination to pembrolizumab in unresectable or metastatic melanoma suggested to reconsider if some elements have been neglected in IDO1 landscape [8].

From a clinical point of view, the best combination regimen, the most responsive patients and the biomarkers that allow to identify them, and the most responsive tumor settings still need to be identified [9]. Additional efforts in the IDO1 field are required even from a medicinal chemistry point of view. A major challenge is the discovery of IDO1 inhibitors with optimal profiles for clinical development, especially in relation to achievement of bioavailability, maintenance of inhibition levels in vivo, crossing of the BBB to target brain metastases, and in-depth functional characterization. Moreover, the correlation between inhibition kinetics and in vivo antitumor activity is still not clear and the data that will arise during clinical trials will help to clarify if it is more effective a competitive, a non-competitive or an irreversible inhibitor. Finally, a better comprehension of the possible interactions with the residues in the IDO1 active site is needed in order to guide the rational design of other novel and potent IDO1 inhibitors. 

## 2. Results

### 2.1. Chemistry

**Figure 1:**
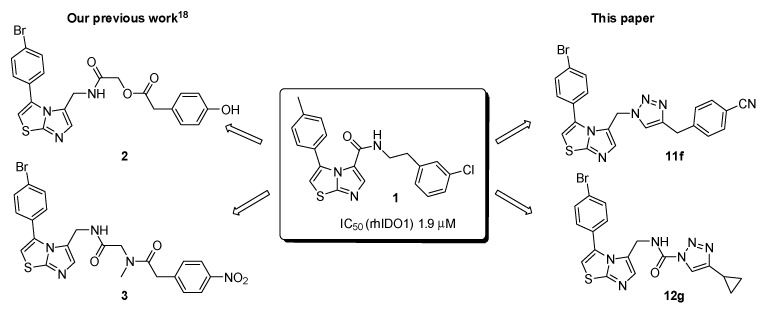
Structure of imidazothiazole compounds **1**, **2**, **3**, **11f** and **12g**.

In 2014, imidazothiazole compounds were reported by Tojo et al. as potent IDO1 inhibitors, with the reference compound **1** (Figure 1) displaying an IC_50_ value of 1.9 µM (rhIDO1) [10]. Taking advantage of our extensive experience both in multicomponent reactions [11,12] and click chemistry approach [13], as well as their exploitation in drug discovery [14,15,16,17], we decided to synthesize in parallel two series of imidazothiazole analogues. 

**Figure 2:**
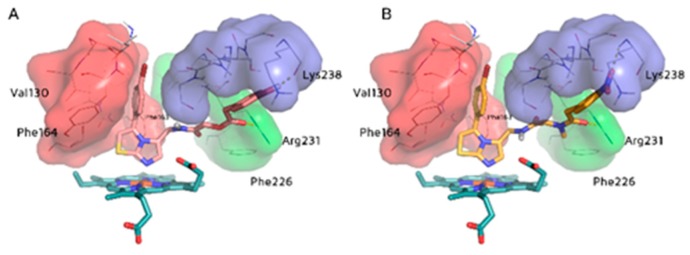
Docking poses of compounds **2** (**A**) and **3** (**B**). Structures are depicted as pink and orange sticks, respectively, while heme is depicted as cyan sticks. Red surface: pocket A; green surface: pocket B; blue surface: pocket C.

The first series, published in 2018 [18], was obtained using Passerini three-component and Ugi four-component reactions. The most potent compounds are represented by **2** and **3** (Figure 1) with IC_50_ values of 0.20 µM and 0.80 µM, respectively. Docking studies have suggested the ability of the imidazole nitrogen to coordinate the iron moiety in the heme group (Figure 2). Furthermore, the long chain protrudes in a region, named pocket C, which has never been considered by previous IDO inhibitors and a crucial hydrogen bond with Lys238 favours the inhibitory activity. 

In the meanwhile, we wondered whether the benzylamide moiety, represented by compounds **2** and **3,** could be isosterically replaced by a 1,4-disubstitued 1,2,3-triazole ring. It must be acknowledged that the triazole moiety has been previously described by Röhrig et al. [19] in IDO1 inhibitors and, more recently, in inhibitors selective for IDO2 [20]. In 2018 other triazole-displaying IDO1 inhibitors were reported [21]. In all the described examples, the nitrogen of the triazole ring makes a bond with the iron of the heme group.

**Scheme 1:**
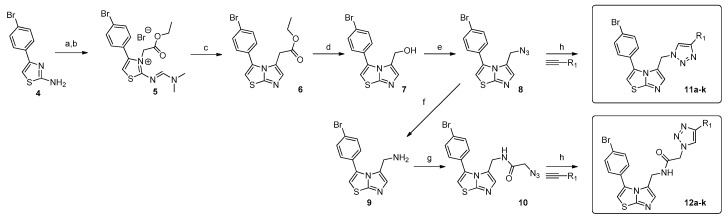
*Reagents and conditions*: (a) DMF-DMA, DMF, 80 °C, 99%; (b) ethyl bromoacetate, 80 °C, 76%; (c) DBU, DMF, 60 °C, 99%; (d) LiAlH_4_, dry THF, 0 °C, 81%; (e) DPPA, DBU, DMF, 60 °C, 67%; (f) PPh_3_, H_2_O, THF, 45 °C, 96%; (g) 2-azidoacetic acid, EDCI, DMAP, TEA, CH_2_Cl_2_, 66%; (h) sodium ascorbate, CuSO_4_•5H_2_O, *t*-BuOH, H_2_O, room temperature (rt), 41–87%.

With the aim to further probe interactions of the side chain of imidazothiazoles with the active site of IDO1 enzyme, and especially with pocket C, we synthesized two classes of imidazothiazoles, the first one starting from azide **8** and the second from azide **10** (Scheme 1). 

Both the imidazothiazole azides can be easily obtained by exploiting a common synthetic route starting from 4-(4-bromophenyl)thiazol-2-amine (**4**) that reacts with DMF-DMA and with ethyl bromoacetate to afford intermediate **5**. Compound **5** undergoes an intramolecular cyclization and a reduction to yield compound **7** that reacts in the presence of DPPA to form the required azide **8**. The latter is reduced via a Staudinger reaction and then compound **9** is coupled with 2-azidoacetic acid to form azide **10** (Scheme 1). 

With the aim to maximize the interaction of the products in IDO1 active site, the synthetic effort was in silico-guided. To this end, alkynes purchasable and/or previously synthesized in our lab were virtually combined with azides **8** and **10** to generate a small library of 88 candidates that were screened in the IDO1 active site. The compounds were then ranked according to their binding energies in the IDO1 protein and the virtual candidates displaying the highest score per each of the two classes were inspected. Finally, the corresponding alkyne was selected for being coupled with both the azides **8** and **10**, for reasons of systematicity and completeness.

Following this approach, 11 alkynes were chosen, and 22 compounds were prepared exploiting the click chemistry reaction under classical conditions, eleven (**11a**–**11k**) from azide **8** and eleven (**12a**–**12k**) from azide **10**. Products precipitated from the reaction mixture and, after filtration, were purified by column chromatography.

### 2.2. Biological Evaluation 

All the 22 synthesized compounds were then biologically evaluated (Table 1). The compounds were tested for their ability to inhibit human IDO1 in an enzyme-based assay using a purified recombinant human IDO1 (rhIDO1) enzyme (Table 1). Each compound (1 µM) was added to the reaction buffer and the rhIDO1 conversion of l-Trp to l-KYN was determined spectrophotometrically using *p*-dimethylaminobenzaldehyde. As reference, imidazothiazole compound 1-((3-(4-bromophenyl)-3a*H*-thieno [2,3-*b*]pyrrol-4-yl)methyl)-3-(4-cyanophenyl)urea [10], displaying an IC_50_ value of 0.08 µM, was chosen. 

In series **11a**–**11k**, among the aromatic substituents on the triazole ring only cyanomethylphenyl ring confers significant inhibitory activity at 1 µM (**11f**, 74%), while within the aliphatic substructures both the propanoyl and the 5-cyanopentanoyl lead to good inhibition (**11j** and **11k**, 81 and 62%, respectively). In series **12a**–**12k** the best activity is given by the cyclopropyl substituent (**12g**, 70%), while benzyl and 1-hydroxycyclohexanyl (**12a** and **12h**) are able to give a 63% of inhibition.

IC_50_ values were determined for compounds that displayed an inhibition above 70% (**11f**, **11j** and **12g**). Compound **11f** has an IC_50_ value of 0.5 ± 0.04 µM, **11j** displays IC_50_ value of 1.1 ± 0.07 µM and compound **12g** of 0.2 ± 0.01 µM.

### 2.3. Molecular Docking Study 

**Figure 3:**
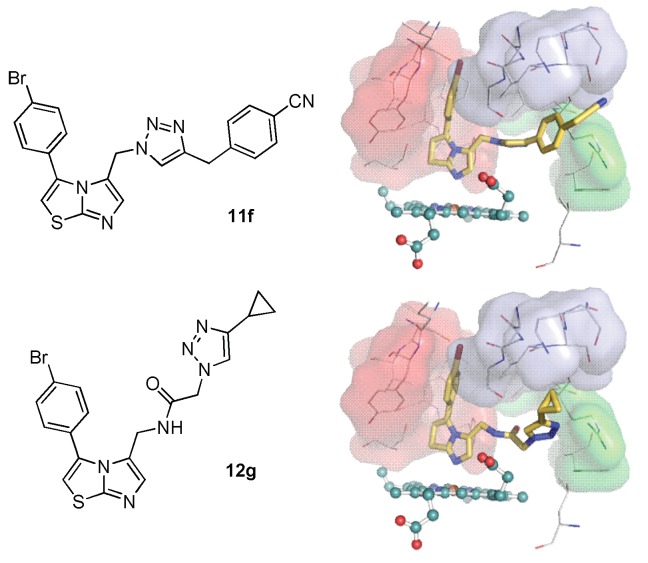
Structure and docking pose of the most potent imidazothiazoles **11f** and **12g**. Structures of docking poses are depicted as gold sticks, while heme is depicted as cyan sticks. Amino acids of pocket A, B and C are depicted as red, green and blue lines shape, respectively.

Molecular modelling was exploited to understand the potential pose of imidazothiazoles in the IDO1 binding site. Docking studies were carried out using the software OMEGA2 [22,23,24] and FRED [25,26], showing that the most potent imidazothiazoles (**11f** and **12g**) (Figure 3) lay with a partially different orientation than compounds **2** and **3** (Figure 2). The *p*-bromophenyl ring of both **11f** and **12g** is accommodated in the hydrophobic pocket A (Tyr126, Cys129, Val130, Phe163 and Phe164), as for compounds **2** and **3**, while the imidazothiazole core is able to form a nitrogen–iron bond with the heme group. The triazole group extends in proximity to pocket B (Phe226 and Arg231) while the side chain derived by alkyne reagent protrudes in the direction of pocket C (Leu234, Ser235, Gly236, Lys238, Ala260, Gly261 and Gly262), without fully occupying this external part of the IDO binding site as shown by **2** and **3**. All the other compounds display similar binding modes (Supporting information Table S1): the interactions in pocket A and B are conserved among all the evaluated structures, while binding to pocket C is more compound-dependent. 

## 3. Materials and Methods 

### 3.1. Chemistry

#### 3.1.1. General Chemistry 

Commercially available reagents and solvents were purchased from Merck (Darmstadt, Germany) and Alfa Aesar (Thermo-Fisher Scientific, Waltham, USA, MA) and used without further purification. Column chromatography was performed on Merck Kieselgel 70–230 mesh ASTM silica gel. Thin layer chromatography (TLC) was carried out on 5 × 20 cm plates with a layer thickness of 0.25 mm (Merck silica gel 60 F254). When necessary, TLC plates were visualized with aqueous KMnO_4_ or with aqueous Pancaldi solution. Melting points were determined in open glass capillary with a SMP3 apparatus (Stuart Scientific, Cole-Parmer, Stone, Staffordshire, UK). All the target compounds were checked by IR (FT-IR Thermo-Nicolet Avatar), ^1^H- and ^13^C-NMR (JEOL ECP 300 MHz, JEOL, Pleasanton, USA, CA), and mass spectrometry (Thermo Finningan LCQ-deca XP-plus) equipped with an ESI source and an ion trap detector. Chemical shifts are reported in parts per million (ppm). The purity of compounds was determined by high performance liquid chromatography (HPLC). Purity of final compounds was 95% or higher. 

#### 3.1.2. Synthesis of 2-azido-*N*-((3-(4-bromophenyl)imidazo[2,1-b]thiazol-5-yl)methyl)acetamide, (**10**)

Amine **9** (700 mg, 2.27 mmol, 1 equiv.) was dissolved in dry CH_2_Cl_2_ (12 mL) under nitrogen atmosphere. 2-Azidoacetic acid (229 mg, 2.27 mmol, 1 equiv.), EDCI (352 mg, 2.27 mmol, 1 equiv.), DMAP (28 mg, 0.23 mmol, 0.1 equiv.) and TEA (316 μL, 2.27 mmol, 1 equiv.) were added in order. The resulting mixture was stirred overnight. When the reaction was finished, CH_2_Cl_2_ was added and the organic layer was washed with water (2×), dried over sodium sulfate and evaporated. The crude material was purified by column chromatography using EtOAc and EtOAc/MeOH 95:5 as eluents, affording 2-azido-*N*-((3-(4-bromophenyl)imidazo[2,1-b]thiazol-5-yl)methyl)acetamide **10** as a white solid (66%). ^1^H-NMR (300 MHz, CD_3_OD): δ 7.68 (d, *J* = 8.2 Hz, 2H), 7.49 (d, *J* = 8.2, 2H), 7.24 (s, 1H), 7.06 (s, 1H), 4.23 (s, 2H), 3.59 (s, 2H).

#### 3.1.3. General Procedure for the Synthesis of Compounds **11a**–**11k** and **12a**–**12k**

To a suspension of azide (0.29 mmol, 1 equiv.) in water (570 µL) and *t*-BuOH (570 µL) alkyne (0.29 mmol, 1 equiv.) was added. Then, 30 µL of aqueous solution of sodium ascorbate 1 M and copper sulfate pentahydrate (0.0029 mmol, 0.01 eq) were added and the mixture was maintained under vigorous stirring overnight. The reaction was normally completed between 3 and 24 h. Ice was added and the precipitate was filtered and rinsed with water and heptane. In some cases (**11a**, **11e**, **11g**, **12a**, **12b**, **12d**, **12e**, **12f**, **12g**, **12i**, **12j**, **12k**) product precipitated and the precipitated solid was collected by filtration using a Buchner funnel and rinsed with ethyl acetate. In case that no precipitation was observed (**11b**, **11c**, **11d**, **11f**, **11h**, **11i**, **11j**, **11k**, **12c**, **12h**) solvent was evaporated and the crude material was purified by column chromatography using the eluent indicated in the analytical data section.

#### 3.1.4. Characterization of Compounds **11a**–**11k** and **12a**–**12k**

*5-((4-Benzyl-1H-1,2,3-triazol-1-yl)methyl)-3-(4-bromophenyl)imidazo[2,1-b]thiazole,* (**11a**). Yellow solid. Yield: 77%. m.p. 106.5–107.5 °C. ^1^H-NMR (300 MHz, DMSO-*d_6_*): δ 7.45 (d, *J* = 6.6 Hz, 2H), 7.30–7.18 (m, 9H), 7.01 (s, 1H), 5.41 (s, 2H), 3.82 (s, 2H). IR (KBr): ṽ = 3113, 2931, 1541, 1455, 1145, 1044, 1013, 844, 815 cm^−1^. MS (ESI) *m*/*z* 451 [M + H]^+^.

*3-(4-Bromophenyl)-5-((4-phenyl-1H-1,2,3-triazol-1-yl)methyl)imidazo[2,1-b]thiazole,* (**11b**). White solid. Yield: 51%. PE/EtOAc 2:8. m.p. 199–199.5 °C dec. ^1^H-NMR (300 MHz, CD_3_OD): δ 7.67 (d, *J* = 6.9 Hz, 2H), 7.50 (s, 1H), 7.47 (d, *J* = 8.5 Hz, 2H), 7.39–7.34 (m, 4H), 7.22 (d, *J* = 8.5 Hz, 2H), 6.99 (s, 1H), 5.51 (s, 2H). IR (KBr): ṽ = 3084, 2923, 2855, 1574, 1458, 1297, 1140, 1070, 755 cm^−1^. MS (ESI) *m*/*z* 437 [M + H]^+^.

*3-(4-Bromophenyl)-5-((4-(4-methoxyphenyl)-1H-1,2,3-triazol-1-yl)methyl)imidazo[2,1-b]thiazole,* (**11c**). Yellow solid. Yield: 87%. PE/EtOAc 3:7. m.p. 118–119 °C dec. ^1^H-NMR (300 MHz, DMSO-*d_6_*): δ 7.63–7.60 (m, 3H), 7.52 (d, *J* = 6.6 Hz, 2H), 7.39 (s, 1H), 7.32 (d, *J* = 7.1 Hz, 2H), 7.14 (s, 1H), 6.97 (d, *J* = 7.1 Hz, 2H), 5.61 (s, 2H), 3.78 (s, 3H). IR (KBr): ṽ = 3018, 2928, 1563, 1459, 1251, 1027, 833, 794 cm^−1^. MS (ESI) *m*/*z* 467 [M + H]^+^.

*4-(1-((3-(4-Bromophenyl)imidazo[2,1-b]thiazol-5-yl)methyl)-1H-1,2,3-triazol-4-yl)aniline,* (**11d**). Yellow solid. Yield: 43%. EtOAc. m.p. 117–118 °C dec. ^1^H-NMR (300 MHz, DMSO-*d_6_*): δ 7.55 (s, 1H), 7.43–7.35 (m, 7H), 7.17 (s, 1H), 6.60 (d, *J* = 5.5 Hz, 2H), 5.46 (s, 2H), 5.14 (br s, 2H). IR (KBr): ṽ = 3329, 3103, 2925, 1729, 1500, 1457, 1294, 836, 763 cm^−1^. MS (ESI) *m*/*z* 452 [M + H]^+^.

*4-(1-((3-(4-Bromophenyl)imidazo[2,1-b]thiazol-5-yl)methyl)-1H-1,2,3-triazol-4-yl)benzonitrile,* (**11e**)**.** Yellow solid. Yield: 83%. m.p. 190–191 °C dec. ^1^H-NMR (300 MHz, DMSO-*d_6_*): δ 8.00 (s, 1H), 7.90–7.88 (m, 5H), 7.47 (d, *J* = 7.7 Hz, 2H), 7.31 (d, *J* = 7.9 Hz, 2H), 7.18 (s, 1H), 5.60 (s, 2H). IR (KBr): ṽ = 3076, 2219, 1928, 1612, 1445, 1147, 844, 803, 555 cm^−1^. MS (ESI) *m*/*z* 462 [M + H]^+^.

*2-(4-(1-((3-(4-Bromophenyl)imidazo[2,1-b]thiazol-5-yl)methyl)-1H-1,2,3-triazol-4-yl)phenyl)acetonitrile,* (**11f**). Yellow solid. Yield: 76%. PE/EtOAc 1:9. m.p. 121.5–122.5 °C. ^1^H-NMR (300 MHz, DMSO-*d_6_*): δ 7.75–7.71 (m, 4H), 7.51 (d, *J* = 7.3 Hz, 2H), 7.40–7.30 (m, 4H), 7.19 (s, 1H), 5.58 (s, 2H), 4.06 (s, 2H). ^13^C-NMR (75 MHz, DMSO-*d_6_*): δ 146.1, 132.1 (3C), 131.7 (2C), 131.2, 130.5, 129.1 (2C), 126.3 (2C), 123.8, 121.2, 119.7, 112.3, 41.2, 22.8. IR (KBr): ṽ = 3100, 2963, 2105, 1487, 1456, 1149, 840, 810, 752 cm^−1^. MS (ESI) *m*/*z* 476 [M + H]^+^.

*3-(4-Bromophenyl)-5-((4-cyclopropyl-1H-1,2,3-triazol-1-yl)methyl)imidazo[2,1-b]thiazole,* (**11g**). Yellow solid. Yield: 44%. m.p. 145.5–146.5 °C. ^1^H-NMR (300 MHz, DMSO-*d_6_*): δ 7.57 (d, *J* = 8.2 Hz, 2H), 7.38 (s, 1H), 7.33 (d, *J* = 8.2 Hz, 2H), 7.18 (s, 1H), 6.97 (s, 1H), 5.38 (s, 2H), 1.76 (quint, *J* = 6.6 Hz, 1H), 0.82 (d, *J* = 6.6 Hz, 2H), 0.55 (d, *J* = 4.6 Hz, 2H). IR (KBr): ṽ = 3012, 2922, 2856, 1733, 1457, 1142, 1041, 1013, 929, 814 cm^−1^. MS (ESI) *m*/*z* 401 [M + H]^+^.

*1-(1-((3-(4-Bromophenyl)imidazo[2,1-b]thiazol-5-yl)methyl)-1H-1,2,3-triazol-4-yl)cyclohexanol,* (**11h**). Yellow solid. Yield: 68%. PE/EtOAc 2:8. m.p. 163.5–164.5 °C dec. ^1^H-NMR (300 MHz, DMSO-*d_6_*): δ 7.58 (d, *J* = 7.9 Hz, 2H), 7.43 (s, 1H), 7.35 (d, *J* = 7.9 Hz, 2H), 7.21 (s, 1H), 7.08 (s, 1H), 5.42 (s, 2H), 4.61 (br s, 1H), 1.75-1.61 (m, 6H), 1.38-1.29 (m, 4H). IR (KBr): ṽ = 3276, 2927, 1573, 1458, 1254, 979, 846, 744 cm^−1^. MS (ESI) *m*/*z* 459 [M + H]^+^.

*3-(1-((3-(4-Bromophenyl)imidazo[2,1-b]thiazol-5-yl)methyl)-1H-1,2,3-triazol-4-yl)propan-1-ol,* (**11i**)**.** Yellow oil. Yield: 45%. EtOAc/MeOH 8:2. ^1^H-NMR (300 MHz, DMSO-*d_6_*): δ ^1^H-NMR (300 MHz, DMSO-*d_6_*): δ 7.55–7.40 (m, 4H), 7.18 (d, *J* = 8.2 Hz, 2H), 6.88 (s, 1H), 5.40 (s, 2H), 5.12 (br s, 1H), 4.42 (t, *J* = 7.4 Hz, 2H), 2.53-2.51 (m, 2H), 1.64 (t, *J* = 7.4 Hz, 2H). IR (neat): ṽ = 3306, 2924, 2854, 1663, 1456, 1088, 922, 888, 701, 603 cm^−1^. MS (ESI) *m*/*z* 419 [M + H]^+^.

*3-(1-((3-(4-Bromophenyl)imidazo[2,1-b]thiazol-5-yl)methyl)-1H-1,2,3-triazol-4-yl)propanoic acid,* (**11j**). Dark yellow solid. Yield: 41%. EtOAc:MeOH 9:1. m.p. 148–148.5 °C dec. ^1^H-NMR (300 MHz, DMSO-*d_6_*): δ 7.42–7.37 (m, 4H), 7.17 (d, *J* = 8.9 Hz, 2H), 7.08 (s, 1H), 5.40 (s, 2H), 2.71 (t, *J* = 6.7 Hz, 2H), 2.45 (t, *J* = 6.7 Hz, 2H). IR (KBr): ṽ = 3180, 2923, 2853, 1715, 1457, 1307, 819, 766 cm^−1^. MS (ESI) *m*/*z* 433 [M + H]^+^.

*5-(1-((3-(4-Bromophenyl)imidazo[2,1-b]thiazol-5-yl)methyl)-1H-1,2,3-triazol-4-yl)pentanenitrile,* (**11k**). Amorphous yellow solid. Yield: 48%. EtOAc. ^1^H-NMR (300 MHz, DMSO-*d_6_*): δ 7.57 (s, 1H), 7.41–7.32 (m, 3H), 7.17 (d, *J* = 8.8 Hz, 2H), 7.07 (s, 1H), 5.42 (s, 2H), 2.60-2.58 (m, 2H), 1.64-1.56 (m, 6H). IR (neat): ṽ = 2932, 2258, 1573, 1454, 1144, 1045, 812, 734 cm^−1^. MS (ESI) *m*/*z* 442 [M + H]^+^.

*2-(4-Benzyl-1H-1,2,3-triazol-1-yl)-N-((3-(4-bromophenyl)imidazo[2,1-b]thiazol-5-yl)methyl)acetamide,* (**12a**)**.** White solid. Yield: 81%. m.p. 177–177.5 °C. ^1^H-NMR (300 MHz, DMSO-*d_6_*): δ 8.34 (br s, 1H), 7.66–7.64 (m, 3H), 7.53 (d, *J* = 7.1 Hz, 2H), 7.30–7.21 (m, 6H), 4.81 (s, 2H), 4.08 (s, 2H), 4.00 (s, 2H). IR (KBr): ṽ = 3027, 2904, 2858, 1676, 1458, 1218, 1143, 1074, 1016, 815, 715 cm^−1^. MS (ESI) *m*/*z* 508 [M + H]^+^.

*N-((3-(4-Bromophenyl)imidazo[2,1-b]thiazol-5-yl)methyl)-2-(4-phenyl-1H-1,2,3-triazol-1-yl)acetamide,* (**12b**)**.** Yellow solid. Yield: 77%. m.p. 251.5-252.5 °C dec. ^1^H-NMR (300 MHz, DMSO-*d_6_*): δ 8.44 (s, 1H), 8.37 (s, 1H), 7.85 (d, *J* = 8.2 Hz, 2H), 7.65 (d, *J* = 7.4 Hz, 2H), 7.56 (d, *J* = 8.2 Hz, 2H), 7.46 (t, *J* = 7.4 Hz, 2H), 7.36 (t, *J* = 7.4 Hz, 1H), 7.22 (s, 1H), 4.93 (s, 2H), 4.09 (s, 2H). IR (KBr): ṽ = 3283, 3063, 2931, 2785, 1652, 1548, 1454, 1149, 1072, 762, 690 cm^−1^. MS (ESI) m/z 494 [M + H]^+^.

*N-((3-(4-Bromophenyl)imidazo[2,1-b]thiazol-5-yl)methyl)-2-(4-(4-methoxyphenyl)-1H-1,2,3-triazol-1-yl)acetamide,* (**12c**)**.** White solid. Yield: 46%. PE/EtOAc 2:8. m.p. 183–184 °C. ^1^H-NMR (300 MHz, DMSO-*d_6_*): δ 8.38 (s, 1H), 8.25 (s, 1H), 7.78 (d, *J* = 8.2 Hz, 2H), 7.68 (d, *J* = 7.9 Hz, 2H), 7.56 (d, *J* = 7.9 Hz, 2H), 7.22 (s, 1H), 7.02 (d, *J* = 8.2 Hz, 2H), 4.92 (s, 2H), 4.08 (s, 2H), 3.80 (s, 3H). IR (KBr): ṽ = 3182, 2924, 1684, 1560, 1454, 1261, 973, 833, 800 cm^−1^. MS (ESI) *m*/*z* 524 [M + H]^+^.

*2-(4-(4-Aminophenyl)-1H-1,2,3-triazol-1-yl)-N-((3-(4-bromophenyl)imidazo[2,1-b]thiazol-5-yl)methyl)acetamide,* (**12d**)**.** Grey solid. Yield: 83%. m.p. 217–218 °C dec. ^1^H-NMR (300 MHz, DMSO-*d_6_*): δ 8.31 (s, 1H), 8.05 (s, 1H), 7.68 (d, *J* = 8.1 Hz, 2H), 7.53-7.50 (m, 4H), 7.16 (s, 1H), 6.64 (d, *J* = 7.4 Hz, 2H), 5.16 (br s, 1H), 4.87 (s, 2H), 4.35 (s, 2H). IR (KBr): ṽ = 3342, 3193, 2931, 1682, 1560, 1502, 1456, 1013, 835, 814 cm^−1^. MS (ESI) *m*/*z* 509 [M + H]^+^.

*N-((3-(4-Bromophenyl)imidazo[2,1-b]thiazol-5-yl)methyl)-2-(4-(4-cyanophenyl)-1H-1,2,3-triazol-1-yl)acetamide,* (**12e**)**.** Yellow solid. Yield: 76%. m.p. 267–267.5 °C dec. ^1^H-NMR (300 MHz, DMSO-*d_6_*): δ 8.60 (s, 1H), 8.47 (s, 1H), 8.07 (d, *J* = 8.2 Hz, 2H), 7.93 (d, *J* = 7.9 Hz, 2H), 7.67 (d, *J* = 8.2 Hz, 2H), 7.56 (d, *J* = 7.7 Hz, 2H), 7.23 (s, 1H), 4.97 (s, 2H), 4.09 (s, 2H). IR (KBr): ṽ = 3298, 3084, 3044, 2223, 1648, 1536, 1454, 1146, 846, 813 cm^−1^. MS (ESI) m/z 519 [M + H]^+^.

*N-((3-(4-Bromophenyl)imidazo[2,1-b]thiazol-5-yl)methyl)-2-(4-(4-(cyanomethyl)phenyl)-1H-1,2,3-triazol-1-yl)acetamide,* (**12f**). Yellow solid. Yield: 68%. m.p. 231–231.5 °C. ^1^H-NMR (300 MHz, DMSO-*d_6_*): δ 8.44 (br s, 1H), 8.42–8.40 (m, 2H), 7.89 (d, *J* = 7.7 Hz, 2H), 7.68 (d, *J* = 7.1 Hz, 2H), 7.55 (d, *J* = 7.1 Hz, 2H), 7.43 (d, *J* = 7.7 Hz, 2H), 7.22 (s, 1H), 4.93 (s, 2H), 4.15 (s, 2H), 4.07 (s, 2H). IR (KBr): ṽ = 3063, 2936, 2244, 1659, 1548, 1455, 1229, 1071, 1013, 820, 794 cm^−1^. MS (ESI) *m*/*z* 533 [M + H]^+^.

*N-((3-(4-Bromophenyl)imidazo[2,1-b]thiazol-5-yl)methyl)-2-(4-cyclopropyl-1H-1,2,3-triazol-1-yl)acetamide,* (**12g**). White solid. Yield: 46%. m.p. 225–225.5 °C dec. ^1^H-NMR (300 MHz, DMSO-*d_6_*): δ 8.35 (s, 1H), 7.68–7.63 (m, 3H), 7.54 (d, *J* = 7.4 Hz, 2H), 7.21 (s, 1H), 4.78 (s, 2H), 4.03 (s, 2H), 1.94 (quint, *J* = 7.1 Hz, 1H), 0.90 (q, *J* = 7.1 Hz, 2H), 0.72 (q, *J* = 7.3 Hz, 2H). ^13^C-NMR (75 MHz, DMSO-*d_6_*): δ 165.1, 146.7, 132.1 (2C), 131.9, 131.3, 129.5, 128.4, 125.7, 123.9, 123.3, 111.9, 51.6, 42.6, 8.2, 7.1. IR (KBr): ṽ = 3104, 2950, 2858, 1681, 1548, 1460, 1449, 1218, 1145, 1014, 818 cm^−1^. MS (ESI) *m*/*z* 458 [M + H]^+^.

*N-((3-(4-Bromophenyl)imidazo[2,1-b]thiazol-5-yl)methyl)-2-(4-(1-hydroxycyclohexyl)-1H-1,2,3-triazol-1-yl)acetamide,* (**12h**). Yellow solid. Yield: 26%. EtOAc/MeOH 9:1. m.p. 165–166 °C. ^1^H-NMR (300 MHz, DMSO-*d_6_*): δ 8.35 (s, 1H), 7.73–7.70 (m, 3H), 7.58 (d, *J* = 7.9 Hz, 2H), 7.23 (s, 1H), 4.85 (s, 2H), 4.06 (s, 2H), 2.00–1.92 (m, 4H), 1.73–1.64 (m, 4H), 1.46–1.42 (m, 2H). IR (KBr): ṽ = 3144, 3043, 2933, 1697, 1558, 1458, 1251, 1071, 846, 809 cm^−1^. MS (ESI) *m*/*z* 516 [M + H]^+^.

*N-((3-(4-Bromophenyl)imidazo[2,1-b]thiazol-5-yl)methyl)-2-(4-(3-hydroxypropyl)-1H-1,2,3-triazol-1-yl)acetamide,* (**12i**). White solid. Yield: 82%. m.p. 169–170 °C dec. ^1^H-NMR (300 MHz, DMSO-*d_6_*): δ 8.30 (s, 1H), 7.68–7.66 (m, 3H), 7.54 (d, *J* = 7.8 Hz, 2H), 7.19 (s, 1H), 4.82 (s, 2H), 4.43 (br s, 1H), 4.17 (s, 2H), 3.46 (t, *J* = 7.1 Hz, 2H), 2.67 (quint, *J* = 7.1 Hz, 2H), 1.76 (t, *J* = 7.1 Hz, 2H). IR (KBr): ṽ = 3250, 3107, 2935, 1679, 1544, 1459, 1219, 1072, 816 cm^−1^. MS (ESI) *m*/*z* 476 [M + H]^+^.

*3-(1-(2-(((3-(4-Bromophenyl)imidazo[2,1-b]thiazol-5-yl)methyl)amino)-2-oxoethyl)-1H-1,2,3-triazol-4-yl)propanoic acid,* (**12j**)**.** White solid. Yield: 42%. m.p. 153–154 °C dec. ^1^H-NMR (300 MHz, DMSO-*d_6_*): δ 8.02 (s, 1H), 7.70–7.67 (m, 3H), 7.53 (d, *J* = 8.0 Hz, 2H), 7.19 (s, 1H), 4.81 (s, 2H), 4.12 (s, 2H), 3.51 (t, *J* = 6.2 Hz, 2H), 2.64-2.58 (m, 2H). IR (KBr): ṽ = 3265, 3056, 1663, 1551, 1457, 1144, 838, 813 cm^−1^. MS (ESI) m/z 490 [M + H]^+^.

*N-((3-(4-Bromophenyl)imidazo[2,1-b]thiazol-5-yl)methyl)-2-(4-(4-cyanobutyl)-1H-1,2,3-triazol-1-yl)acetamide,* (**12k**). White solid. Yield: 55%. m.p. 179.5–180.5 °C. ^1^H-NMR (300 MHz, DMSO-*d_6_*): δ 8.32 (s, 1H), 7.70–7.67 (m, 3H), 7.56 (d, *J* = 7.1 Hz, 2H), 7.22 (s, 1H), 4.85 (s, 2H), 4.09 (s, 2H), 2.69 (t, *J* = 6.1 Hz, 2H), 1.69-1.64 (m, 6H). IR (KBr): ṽ = 3280, 2929, 2244, 1680, 1655, 1547, 1452, 1219, 1014, 817 cm^−1^. MS (ESI) *m*/*z* 499 [M + H]^+^.

### 3.2. Biology: rhIDO1 Enzymatic Assay

rhIDO1 activity was determined as follows. In brief, the standard reaction mixture (200 μL) contained 50 mM potassium phosphate buffer (KPB) (pH 6.5), 20 mM ascorbic acid (neutralized with NaOH and HCl) (Sigma Aldrich), 100 μg/mL catalase (Sigma Aldrich), 10 μM methylene blue (Alfa Aesar, Heysham, Lancashire, UK), 100 µM l-tryptophan (Sigma Aldrich), 50 nM rhIDO1 (Origene, Bologna, Italy), and dimethyl sulfoxide (DMSO) solution of the compound (4 μL). The reaction was carried out at 37 °C for 60 min and stopped by the addition of 40 μL of 30% (w/v) CCl_3_COOH. After heating at 50 °C for 15 min, the reaction mixture was centrifuged at 1500× g for 10 min. The supernatant (150 μL) was transferred into a well of a 96-well microplate and mixed with 150 μL of 2% (w/v) p-dimethylaminobenzaldehyde (Ehrlich’s reagent) in acetic acid. The yellow pigment derived from kynurenine was measured at 490 nm using an Ultramark Microplate Imaging System (Bio-Rad, Hercules, CA, USA). IC_50_ values were calculated from concentration-response curves obtained in at least three different experiments run in triplicate.

### 3.3. Docking Study

Protein preparation. Crystal structure of human IDO1 in complex with compound **1** (PDB ID: 4PK6) [10], resolved using X-ray diffraction method with a resolution of 3.45 Å, was retrieved from Protein Data Bank (www.rcsb.org). Despite other X-ray crystal structures have been reported with a better resolution value, the considered crystal structure has a different conformation of the aminoacids in the binding site (mostly in pocket B) that, as already reported, can better accommodate the parental compounds **2** and **3,** since they were designed on structure 1, the co-crystalized compound in PDB 4PK6. Retrieved structure has been further modified for docking calculations as follows: compound **1** was removed, polar hydrogens were added to the protein complex and the resulting polar hydrogens were optimized using the MolProbability server (Durham, NC, USA) [27].

Compound preparation. Alkynes available in house (44 compounds) were considered and virtually combined with azides **8** and **10**. The generated virtual library (88 compounds) was prepared according to our previously reported docking procedure [17], 3D conformations of compounds were generated with the program OMEGA2 (version 2.4.6, Openeye, Santa Fe, NM, USA) [22,23,24].

Docking procedure. The FRED software (version 3.0.0, Openeye) was used in order to dock the compounds in the IDO1 binding site [25,26]. The center of the binding pocket considered in the study was set in the geometrical center of the original co-crystalized compound (**1**), standard setting of FRED was used and the Chemgauss4 scores were considered to evaluate the docking poses. The representation of protein structures and docking results were generated using PyMOL software (version 2.1, New York, NY, USA) [28].

## 4. Conclusions

In conclusion, following a common synthetic route, two series of imidazothiazoles have been synthesized by a click chemistry approach. Three compounds show inhibitory activity on IDO1 in the enzyme-based assay in the low micromolar range. Compound **12g** displays a ten-fold higher inhibitory activity (IC_50_ = 0.2 μM) compared to the starting compound (IC_50_ = 1.9 μM). Moreover, it is characterized by a peculiar binding mode that sees the side chain protruding into an additional pocket, named C and located in the most external part of the IDO binding site. Unfortunately, and similarly to what was observed in the case of imidazothiazoles reported by Tojo [10] and compounds **2** and **3** discovered in our laboratory [18], these molecules are not able to significantly permeate the cell and to inhibit IDO1 in a cell-based assay (data not shown).

Overall, the information acquired both in this study and in our previous work [18] provides new insights in the field of IDO1 inhibitors. Indeed, we have demonstrated that pocket C can be exploited in the design of next generations of IDO1 inhibitors. The integration of this information in a structure-based virtual screening performed in our laboratory has recently led to the identification of structurally novel IDO1 inhibitors with cellular potency in the low nanomolar level and improved clinical potential. These further studies will be reported in due course.

## Figures and Tables

**Table 1:** Structure and biological profile of imidazothiazoles.

		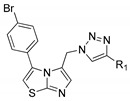		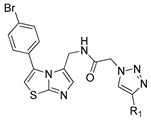	
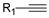		Cpd, Yield (%)	Enzymatic Assay Inhibition (%) at 1 µM ^a^	Cpd, Yield (%)	Enzymatic Assay Inhibition (%) at 1 µM ^a^
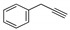	**a**	**11a**, 77%	30 ± 6.7	**12a**, 81%	63 ± 4.3
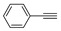	**b**	**11b**, 51%	35 ± 2.7	**12b**, 77%	61 ± 5.5
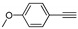	**c**	**11c**, 87%	57 ± 13	**12c**, 46%	5 ± 1.2
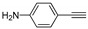	**d**	**11d**, 43%	17 ± 1.2	**12d**, 83%	56 ± 4.7
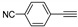	**e**	**11e**, 83%	28 ± 1.5	**12e**, 76%	50 ± 4.5
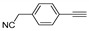	**f**	**11f**, 76%	74 ± 5.8	**12f**, 68%	55 ± 7.2
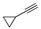	**g**	**11g**, 44%	61 ± 7.1	**12g**, 46%	70 ± 9.2
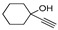	**h**	**11h**, 68%	35 ± 2.4	**12h**, 26%	63 ± 4.1
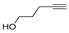	**i**	**11i**, 45%	0	**12i**, 82%	45 ± 2.8
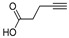	**j**	**11j**, 41%	81 ± 7.9	**12j**, 42%	33 ± 4.5
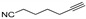	**k**	**11k**, 48%	62 ± 5.6	**12k**, 55%	50 ± 11

^a^ the values are the mean ± SE of two independent experiments.

## Supplementary Materials

The following are available online. Table S1: Structure and biological profile of compounds evaluated by molecular docking.
