# Supplementary material for: Synthesis, Docking and Biological Evaluation of a Novel Class of Imidazothiazoles as IDO1 Inhibitors
Source: Molecules. 2019 May 15;24(10):1874. doi: 10.3390/molecules24101874 (PMC6572114; doi:10.3390/molecules24101874)
Supplement: Supplementary file 1 [file molecules-24-01874-s001.pdf]

Supplementary material

Synthesis, Docking and Biological Evaluation of a Novel Class of Imidazothiazoles as IDO1 Inhibitors

Marta Serafini [1,†], Enza Torre [1,†], Silvio Aprile [1], Alberto Massarotti [1], Silvia Fallarini [1] and Tracey Pirali [1,*]

[1] Department of Pharmaceutical Sciences; Università del Piemonte Orientale, Largo Donegani 2, 28100, Novara, Italy; marta.serafini@uniupo.it (M.S.)

* Correspondence: tracey.pirali@uniupo.it; Tel.: +39-0321-375-852 (T.P.)

† These authors contributed equally to this work.

Table S1. Structure and biological profile of compounds evaluated by molecular docking. Structures of docking poses are depicted as gold sticks, while heme is depicted as cyan sticks. Aminoacids of pocket A, B and C are depicted as red, green and blue shape, respectively.

Cpd	FRED Chemgauss4 score	Docking pose
**11a**	-15.45	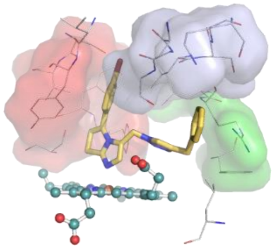
**11b**	-13.56	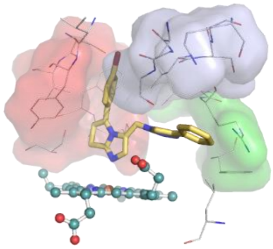
**11c**	-13.99	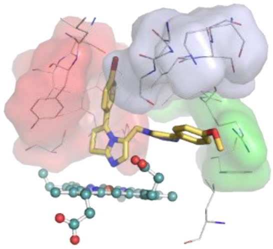
**11d**	-13.97	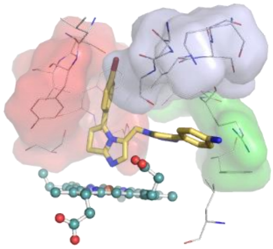
**11e**	-14.89	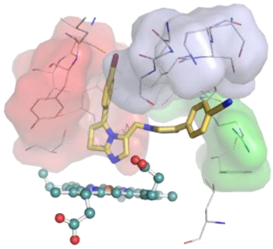

Cpd	FRED Chemgauss4 score	Docking pose
**11f**	-14.36	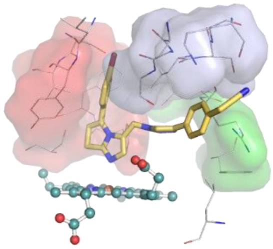
**11g**	-14.86	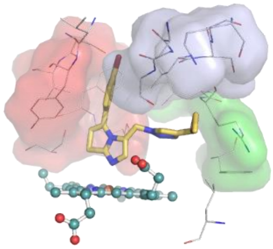
**11h**	-14.40	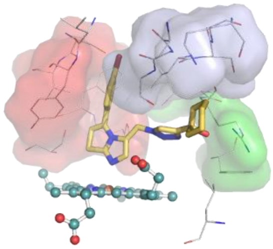
**11i**	-14.67	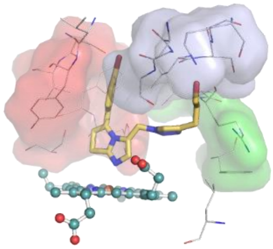
**11j**	-14.18	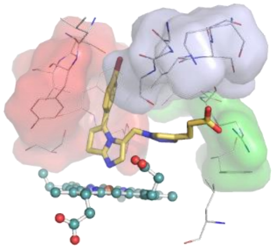

Cpd	FRED Chemgauss4 score	Docking pose
**11k**	-14.35	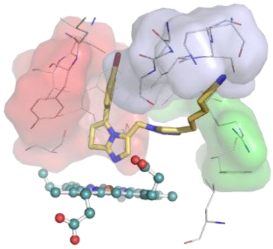
**12a**	-11.98	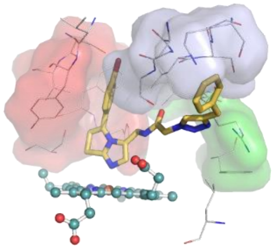
**12b**	-11.70	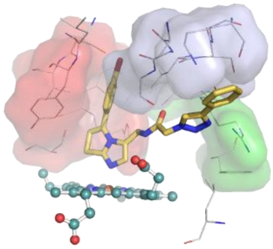
**12c**	-12.38	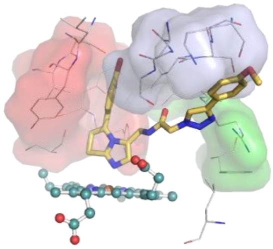
**12d**	-12.39	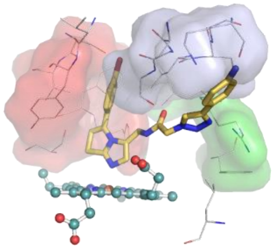

Cpd	FRED Chemgauss4 score	Docking pose
**12e**	-12.74	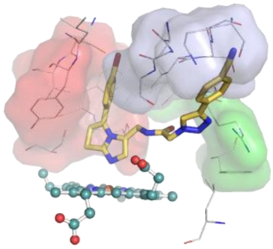
**12f**	-14.46	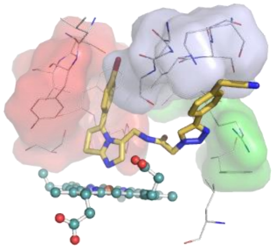
**12g**	-14.87	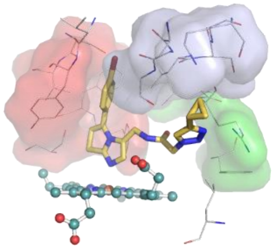
**12h**	-15.99	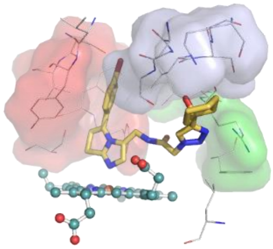
**12i**	-15.16	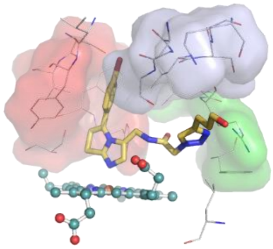

Cpd	FRED Chemgauss4 score	Docking pose
**12j**	-15.31	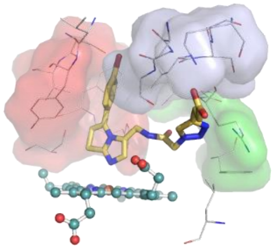
**12k**	-15.37	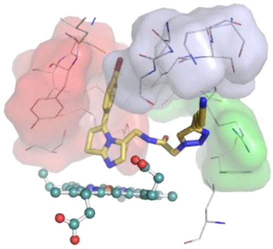
